# Hepatoprotective Effect of *Pericarpium zanthoxyli* Extract Is Mediated via Antagonism of Oxidative Stress

**DOI:** 10.1155/2020/6761842

**Published:** 2020-07-09

**Authors:** Sang Mi Park, Jae Kwang Kim, Eun Ok Kim, Kyung Hwan Jegal, Dae Hwa Jung, Sang Gon Lee, Il Je Cho, SeungMo Kim, Sung Hui Byun, Sae-kwang Ku, Chung A. Park, Chul Won Lee, Won G. An, Sang Chan Kim, Rongjie Zhao

**Affiliations:** ^1^College of Korean Medicine, Daegu Haany University, Gyeongsan 38610, Republic of Korea; ^2^HaniBio Co. Ltd., Gyeongsan 38540, Republic of Korea; ^3^Kabsan Korean Hospital, Seoul 06362, Republic of Korea; ^4^Research Institute for Korean Medicine, Pusan National University, Yangsan 50612, Republic of Korea; ^5^School of Korean Medicine, Pusan National University, Yangsan 50612, Republic of Korea; ^6^Department of Psychopharmacology, Qiqihar Medical University, Qiqihar 161006, China

## Abstract

*Pericarpium zanthoxyli* has been extensively used in traditional Oriental medicine to treat gastric disorders and has anti-inflammatory and antioxidative activities. Therefore, the present study examined a possible hepatoprotective effect of a *P. zanthoxyli* extract (PZE) and investigated the underlying molecular mechanisms. We employed an *in vitro* model of arachidonic acid (AA) + iron-induced hepatocyte damage and an *in vivo* model of CCl_4_-induced liver injury to assess the effects of PZE and evaluated the relevant molecular targets using biochemical assays, flow cytometry analysis, Western blot, and histopathological analysis. The PZE inhibited AA + iron-induced hepatotoxicity in HepG2 cells, improved mitochondrial dysfunction, and reversed an increase in the cellular H_2_O_2_ production and a decrease in the reduced GSH levels induced by AA + iron. Treatment with either 30 or 100 *μ*g/ml PZE significantly increased the expression of nuclear factor erythroid 2-related factor 2 (Nrf2) protein, and the latter dose also increased the antioxidant response element- (ARE-) driven luciferase activity and enhanced the protein expressions of glutamate-cysteine ligase catalytic subunit and NAD(P)H:quinone oxidoreductase 1. In addition, treatment with 100 *μ*g/ml PZE for 3 or 6 h increased the phosphorylation rates of Nrf2 and the extracellular signal-regulated kinase. In the *in vivo* experiment, oral treatment with both 100 and 300 mg/kg PZE inhibited the plasma aspartate aminotransferase activity, and the latter also inhibited the plasma alanine aminotransferase activity. In addition, both doses of PZE ameliorated the parenchymal degeneration and necrosis in the liver induced by CCl_4_ administration, which was associated with reduced expressions of cleaved caspase-3, cleaved poly (ADP-ribose) polymerase, nitrotyrosine, and 4-hydroxynonenal by PZE. These findings suggest that PZE has protective effects against hepatotoxicity both *in vitro* and *in vivo*, which are mainly mediated via its antioxidant activity.

## 1. Introduction

Nowadays, liver disease is still one of the biggest health problems worldwide [1]. Not only is acute liver injury associated with high mortality rates, but it also initiates severe hepatic damage such as liver fibrosis, hepatic cirrhosis, and liver cancer [2]. Excessive oxidative stress caused by stressful events has been shown to be the mechanism underpinning the pathophysiological process in various acute liver injuries [3], highlighting the possibility that that counteracting hepatic oxidative stress can improve both acute liver damage and severe chronic hepatic diseases. Consequently, medical agents with hepatoprotective potential are usually determined for their effects on hepatic oxidative stress [4].

Fortunately, there are several *in vitro* and *in vivo* experimental models available to evaluate drug's effects on hepatic oxidative stress. For example, *in vitro,* simultaneous application of arachidonic acid (AA) and iron to the cultured hepatic origin cells (i.e., HepG2 cells) is widely used to induce hepatic oxidative stress. AA is a polyunsaturated fatty acid that is released from membranes and promotes oxidative stress, apoptosis, necrosis, and inflammatory response [5–7]. In addition, surplus iron catalyzes the release of AA by altering membrane phospholipids [8, 9]; moreover, it synergizes with AA to induce mitochondrial damage and oxidative stress; thereby, AA + iron is typically toxic to hepatocytes [6]. Carbon tetrachloride (CCl_4_) is a hepatotoxicant used widely in animals to investigate *in vivo* the effects of hepatoprotective drugs on toxicant-induced liver injury. CCl_4_ is mainly metabolized in the liver by cytochrome P450 2E1, producing directly or indirectly a variety of free radical metabolites such as trichloromethyl, trichloromethyl peroxyl, and peroxynitrite, which further generate reactive oxygen species (ROS), constituting the molecular basis for the CCl_4_-induced hepatotoxicity [10]. Excessive ROS launch lipid peroxidation of the cellular membrane and endoplasmic reticulum and create disturbance of membrane permeability, reduction of protein synthesis, and impairment of DNA, eventually leading to hepatic degeneration and necrosis [11].

An excessive oxidative stress is fundamentally the result of the imbalance of prooxidant and antioxidant functioning in the tissue; that is to say, antagonization of oxidative stress can be implemented by boosting the antioxidant capacity in the tissue. A transcription factor named nuclear factor erythroid 2-related factor 2 (Nrf2) appears to be an important antioxidant molecule in cells [12, 13]. Nrf2 is a basic leucine zipper protein that initiates the expression of antioxidant proteins which protect against the oxidative damage triggered by endogenous and exogenous toxicants [14]. Nrf2 can be detected in a wide range of tissues, including in the liver. Accordingly, the role of Nrf2 in the liver disorders has been frequently evaluated to identify therapeutic candidates [4].

*Pericarpium zanthoxyli* is the dried pericarp of the ripe fruit from *Zanthoxylum bungeanum* Maxim. or *Zanthoxylum schinifolium* Siebold and Zucc. (Rutaceae), which are distributed in China, Japan, and Korea. Extracts of *P. zanthoxyli* (PZE) have been empirically used in traditional Oriental medicine for treating cold perspiration of the stomach and spleen, stomach pain, indigestion, diarrhea, gastritis, and toothache [15–17]. *P. zanthoxyli* contains many biologically active constituents, such as (−)-aromadendrene, (−)-isopulegol, (+)-gamma-cadinene, (+)-beta-pinene, (−)-N-acetylanonaine (R-type), hydroxyl-*γ*-sanshool, hydroxy-*α*-sanshool, hydroxy-*β*-sanshool, linalool, nerol, zanthoxylin, zanthobungeanine, *α*-pinene, piperitone, skimmianine, *β*-sitosterol, *γ*-sanshool, terpinen-4-ol, (+, −)*α*-sanshool, *α*-terpineol, *α*-thujene, *β*-sanshool, and *trans*-ocimene [18]. Modern scientific experiments have revealed that *P. zanthoxyli* has antiparasitic, antibacterial, anti-inflammatory, antioxidative, and antidiabetic effects [19]. For example, PZE lowered the plasma levels of IL-1*β*, cyclooxygenase-2 (COX-2), and TNF-*α* in rats with cervical spondylotic radiculopathy [20]; flavonoids from *P. zanthoxyli* effectively scavenged hydroxyl free radicals in an *in vitro* experiment [21]; PZE inhibited lipid peroxidation induced by lipopolysaccharide in macrophage RAW 264.7 cells and suppressed the expressions of inducible nitric oxide synthase and COX-2 [22]. These preclinical facts along with the empirical use of *P. zanthoxyli* in treating human digestive diseases prompt us to hypothesize that *P. zanthoxyli* has hepatoprotective effects which may be mediated via its antioxidant properties.

To test this hypothesis, in the present study, we examined whether PZE protects hepatocytes against AA plus iron-induced oxidative stress by manipulating mitochondrial dysfunction, modulating glutathione (GSH) levels and H_2_O_2_ production, and interfering with the apoptotic process; in addition, we examined whether this cytoprotective effect is linked to the induction of antioxidant genes through ERK-mediated Nrf2 signaling. Moreover, in *in vivo* experiments, the possible hepatoprotective effect of PZE was also determined in CCl_4_-treated mice by measuring the plasma activities of the marker enzymes for hepatic functioning and by analyzing histomorphometrically the histopathological profiles of the hepatic damage.

## 2. Materials and Methods

### 2.1. Reagents and Antibodies

AA was obtained from Calbiochem (San Diego, CA, USA). Antibodies against procaspase-3, cleaved caspase-3, poly (ADP-ribose) polymerase (PARP), Bcl-2, lamin A/C, ERK1/2, phospho-ERK1/2, and NAD(P)H:quinone oxidoreductase 1 (NQO1) along with horseradish peroxidase-conjugated goat anti-mouse antibodies were provided by Cell Signaling Technology (Beverly, MA, USA). Anti-Nrf2 and anti-cleaved PARP antibodies were purchased from Santa Cruz Biotechnology (Santa Cruz, CA, USA). The anti-phospho-Nrf2, anti-glutamate-cysteine ligase catalytic subunit (GCLC), and anti-4-hydroxynonenal (4-HNE) polyclonal antibodies were purchased from Abcam (Cambridge, MA, USA). The Fugene® HD and luciferase assay kit were obtained from Promega (Madison, WI, USA). Anti-nitrotyrosine (NT) polyclonal antibody was purchased from Millipore Corporation (Bedford, MA, USA). The 3-(4,5-dimethylthiazol-2-yl)-2,5-diphenyl-tetrazolium bromide (MTT), rhodamine 123 (Rh123), 2′,7′-dichlorofluorescein diacetate (DCFH-DA), silymarin (SIL), anti-*β*-actin antibody, and other reagents were supplied by Sigma-Aldrich (St. Louis, MO, USA).

### 2.2. Preparation of PZE

**Figure 1:**
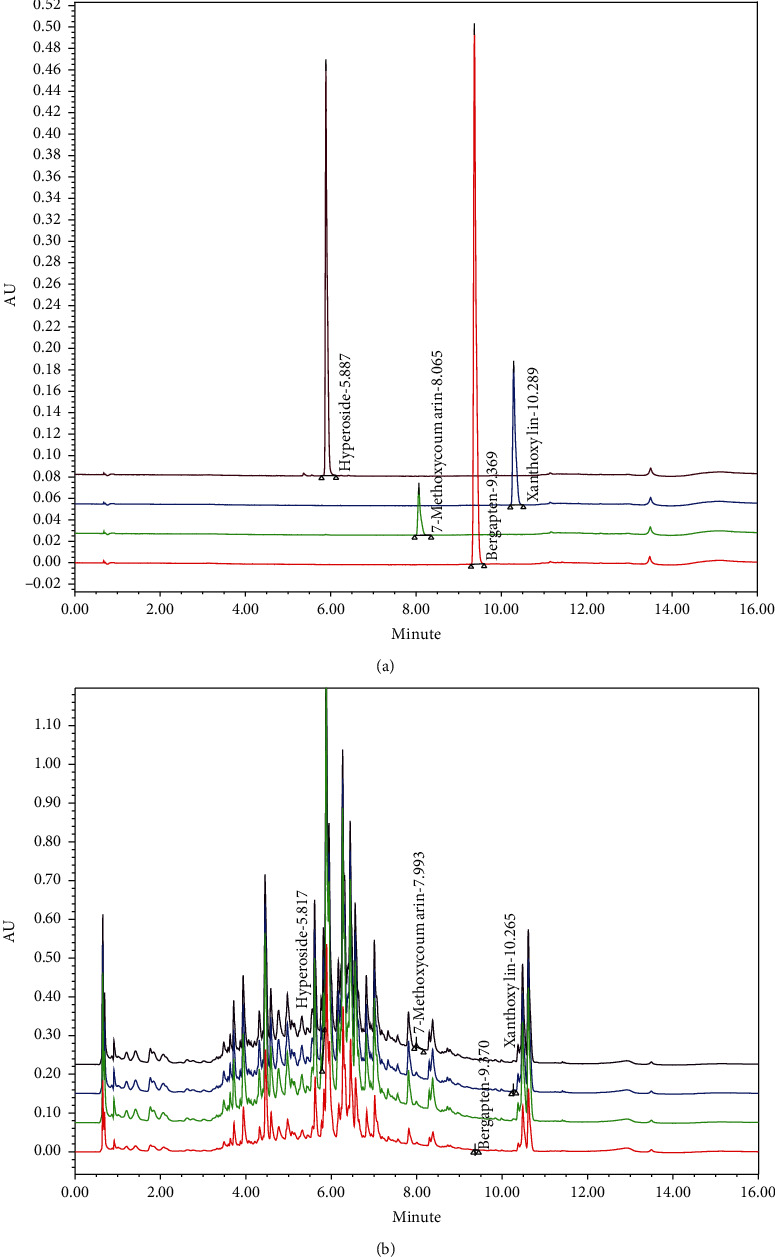
UPLC chromatogram of four marker compounds in PZE. UPLC chromatogram of (a) commercial standard compounds. UPLC chromatogram of (b) four marker compounds in PZE. The chromatograms were obtained at 280 nm (7-methoxycoumarin), 290 nm (xanthoxylin), and 330 nm (bergapten and hyperoside).

*Pericarpium zanthoxyli* was obtained from Daewon Pharmacy (located in Daegu, Republic of Korea), and a voucher specimen (number: DHU-GHFM78) was stored in Daegu Haany University (Republic of Korea). PZE was prepared by extracting 100 g of *P. zanthoxyli* in 1.2 liter of boiling water for 3 h. The PZE was filtered using a 0.22 *μ*m filter (Nalgene, Rochester, NY, USA), lyophilized by a vacuum evaporator, and deposited at −20°C until use. The yield of lyophilized PZE was 14.5%. The ultra-performance liquid chromatography (UPLC) profile of PZE was analyzed using a Waters ACQUITY UPLC system (Waters Corp., Milford, MA, USA) with a Waters ACQUITY photodiode array detector and Waters ACQUITY BEH C18 column (1.7 *μ*m, 2.1 mm × 100 mm) assisted by the Empower software (Figure 1). The PZE includes hyperoside (21.047 ± 0.53 *μ*g/g), 7-methoxycoumarin (19.395 ± 0.63 *μ*g/g), bergapten (2.816 ± 0.096 *μ*g/g), and xanthoxylin (1.756 ± 0.043 *μ*g/g).

### 2.3. Cell Culture

The HepG2 cell line (a human hepatoma cell line) was provided by American Type Culture Collection (Rockville, MD, USA). HepG2 cells were cultured using Dulbecco's modified Eagle's medium (DMEM; Invitrogen, Carlsbad, CA, USA) at 37°C in a humidified 5% CO_2_ atmosphere, and the DMEM contained 10% fetal bovine serum, 50 mg/ml streptomycin, and 50 units/ml penicillin. Cells were incubated in medium without 10% fetal bovine serum for 12 h, stimulated by 10 *μ*M AA for 12 h, and then continued to be incubated with 5 *μ*M iron for the designated time periods. AA was dissolved in dimethyl sulfoxide (DMSO), and iron solution (iron-nitrilotriacetate in NaCl, pH 7.4 adjusted with NaHCO_3_ solution) was prepared before being added to the cells [7]. To determine the effects of PZE, the cells were treated with 10–300 *μ*g/ml PZE (dissolved in distilled water) 1 h prior to the AA exposure. Another set of HepG2 cells undergoing the same experimental schedule with the PZE treated cells were treated with respective vehicles and assigned as the control group.

### 2.4. MTT Assay for Cell Viability

HepG2 cells were cultured with DMEM in a 24-well plate, and the density was 5 × 10^4^ cells per well. The cells were incubated with 0.25 mg/ml MTT for 120 min following treatment with AA + iron, PZE, or their combination. The media were removed, the formazan crystals were dissolved in DMSO, and the absorbance was read at 570 nm with the Tecan Infinite M200 microplate reader (Männedorf, Switzerland). The relative cell viability was analyzed by the following equation:

(1) $$\begin{array}{c} \text{cell viability}=\frac{\text{absorbance of treated sample}}{\text{absorbance of control}}\times 100. \end{array}$$

### 2.5. Measurement of H_2_O_2_ Production

DCFH-DA permeates into the cells and turns into the fluorescent dichlorofluorescein by interacting with H_2_O_2_. The production of H_2_O_2_ was evaluated by detecting the fluorescence intensity of the treated cells after being incubated with DCFH-DA (10 *μ*M) for 60 min at 37°C [7]. The fluorescence intensity was read at an excitation/emission wavelength (485/530 nm) with the Tecan Infinite M200 microplate reader (Tecan).

### 2.6. Determination of Reduced GSH Concentration

The GSH content in HepG2 cells was monitored using a two-step chemical GSH #400 kit (Oxis International, Portland, OR, USA). After the indicated treatments, the cells were lysed in metaphosphoric acid, and the level of reduced GSH was examined by the Tecan Infinite M200 microplate reader (Tecan).

### 2.7. Determination of Mitochondrial Membrane Potential (MMP)

The MMP was examined by employing the Partec GmbH FACSCalibur flow cytometer (Münster, Germany) [7]. The treated cells were stained with Rh123 (0.05 *μ*g/ml), a membrane-permeable cationic fluorescent dye, for 30 min and harvested by trypsinization. Afterward, the cells were suspended in phosphate-buffered saline containing 1% fetal bovine serum and then went through the FACS. In each analysis, 20,000 events were monitored.

### 2.8. Western Blot Analysis

Preparation of whole cell lysates and Western blot analysis were carried out as previously described [23]. HepG2 cells were lysed in RIPA buffer that contained Halt protease and phosphatase inhibitor cocktail (Thermo Scientific) for ten minutes and then centrifuged at 4°C, 15,000 ×g, for thirty minutes. The obtained supernatant was collected, and the total proteins were equally loaded onto SDS-PAGE gels and transferred electrophoretically to a nitrocellulose membrane. The membrane was incubated with the primary antibody and then with the secondary antibody. The interested protein bands were visualized with the assistance of enhanced chemiluminescence (Amersham Biosciences, Buckinghamshire, UK). The *β*-actin immunoblotting was done to prove the equal protein loading. Densitometric analysis was performed with the ImageJ software.

### 2.9. Transient Transfection and Luciferase Reporter Assay

Antioxidant response element- (ARE-) driven reporter gene construct, pGL4.37[luc2P/ARE/Hygro], was provided by Promega. To generate glutathione-S-transferase (GST) A2 promoter-driven luciferase construct, pGL415-rGSTA2-1128, rGSTA2 promoter region from −1128 to −1 bp was amplified by templating pGL3-rGSTA2-1128 and ligated into the KpnI/BglII site of pGL4.15 (Promega). The nucleotide sequence of the construct was proved by sequence analysis with an ABI7700 DNA cycle sequencer. HepG2 cells were transfected by pGL4.37 or pGL415-rGSTA2-1128 plasmid with Fugene HD (Promega) following the manufacturer's instruction, and the selection of the resistant colonies was done by adding 80 *μ*g/ml hygromycin, pooled for the reporter gene analysis. To measure luciferase activity, the transfected cells (5 × 10^5^ cells/well) were replated in 12-well plates overnight, serum-starved for 12 h, and treated with 10–100 *μ*g/ml PZE for 24 h. Luciferase activities in cell lysates were determined using a microplate reader after adding luciferase assay reagent. The relative luciferase activity was calculated based on the protein content that was measured by the BCA method.

### 2.10. CCl_4_-Induced Liver Injury

Six-week-old male ICR mice (30–32 g) were obtained from Orient Bio Inc. (Seongnam, Gyeonggi-do, Korea) and acclimatized for 7 days. CCl_4_ (0.5 ml/kg/day, dissolved in corn oil, 10% vol/vol) was intraperitoneally (i.p.) administered to the mice (5 mice per group) for 2 days to induce liver damage. PZE was dissolved in distilled water and orally (p.o., by gavage) given to the mice at doses of 100 or 300 mg/kg/day for 4 consecutive days. On the first day, the mice were given PZE and, 90 min later, received the first injection of CCl_4_; on the second day and the third day, the mice received PZE treatment without CCl_4_; on the fourth day, the mice were administered the second CCl_4_ 90 min after the fourth PZE treatment. Twenty-four hours after the second CCl_4_, the mice were sacrificed by euthanasia with CO_2_ asphyxiation, and the liver and blood samples were harvested. SIL (100 mg/kg/day) was employed as a positive control of pharmacotherapy. Additionally, a separate group of mice (*n* = 5) treated with the respective vehicles (in the same volume) underwent the same experimental schedule with the PZE treated mice and served as the (vehicle-treated) control group. All animal procedures were approved by the Institutional Animal Care and Use Committee of Daegu Haany University (Approval number: DHU2016-073) and performed in accordance with the national regulations regarding the usage and welfare of laboratory animals.

### 2.11. Blood Biochemistry

Plasma was separated from the blood sample, and alanine aminotransferase (ALT) and aspartate aminotransferase (AST) in plasma were examined with an automated blood analyzer (FUJI DRI-CHEM NX500I, Fuji Medical Systems Co., Ltd., Tokyo, Japan).

### 2.12. Histological Examination

Tissue samples were separated from the left lateral lobe of the liver, fixed in formalin (10%), embedded in paraffin, sectioned (3-4 *μ*m), stained by hematoxylin and eosin (HE) [23, 24], and observed under a light microscope (Eclipse 80*i*, Nikon, Tokyo, Japan). The histopathological profile of each sample includes confluent necrosis, focal lytic necrosis, apoptosis, and focal and portal inflammation, which was assessed by the modified histological activity index (HAI) and graded according to a semiquantitative histopathological scoring system established previously [25] (Table 1). The percentage of degenerative regions (%/mm^2^) in the hepatic sample marking centrolobular necrosis, congestion, and inflammatory cell infiltration was calculated using a computer-assisted automated image analyzer (*i*Solution FL version 9.1, IMT *i*-Solution Inc., Vancouver, BC, Canada), and the hepatocytes with degenerative changes (necrosis, acute cellular swelling, and severe fatty change) were counted. The number of infiltrated inflammatory cells was calculated with an automated image analyzer denoted by cells/1000 hepatocytes, and the number of cells/mm^2^ of hepatic parenchyma was presented according to previous methods [23, 24].

### 2.13. Immunohistochemistry

The cleaved caspase-3, cleaved PARP, 4-HNE, and NT immunoreactive cells were, respectively, detected by an immunohistochemical method using purified primary antibodies, the avidin-biotin-peroxidase, and peroxidase substrate kits (Vector Labs, Burlingame, CA, USA) as described previously [23, 24, 26]. Briefly, the activity of endogenous peroxidase was blocked by incubating the sections in methanol and 0.3% H_2_O_2_ for 0.5 h, and the blockade of nonspecific immunoglobulin binding was implemented with a normal horse serum blocking solution for 60 min in a humidity chamber. The sections were treated with (or without) the primary antisera overnight at 4°C in the humidified chamber, followed by incubation with the biotinylated secondary antibody and ABC reagents for 60 min at room temperature. Then, the sections were allowed to react with the peroxidase substrate kit for 3 min at room temperature. Finally, all sections were rinsed with 0.01 M phosphate-buffered saline for three times; the sections treated without any primary antibodies were considered as the blank controls for the immunohistochemical staining (data not shown); and the sections were observed under the microscope with an aid from the computer-based automated image analyzer (iSolution FL ver 9.1, IMT i-Solution Inc., Burnaby, BC, Canada). Hepatic tissue images in the sections were adjusted to contain 1000 hepatocytes on the view field of the analyzer, which were located in a restricted hepatic parenchyma area around a centrolobular region and central veins. The cells with immunostained (3,3′-diaminobenzidine staining, brown in color) areas over 20% were considered as positive for cleaved caspase-3, cleaved PARP, NT, and 4-HNE, respectively, and the numbers of the positive cells (/1000 hepatocytes) were counted; these processes were all done by two trained observers who were blind to the treatments [23, 24].

### 2.14. Statistical Analysis

All data are expressed as means ± SD (standard deviation). All the data, except for the histomorphometric values, were statistically analyzed with one-way analysis of variance (ANOVA) followed by the Newman–Keuls post hoc test. The homogeneity of variance was checked using the Levene test [27]. The histomorphometric data (*n* = 5) were statistically analyzed by either ANOVA + the least significant difference (LSD) post hoc test or the Kruskal–Wallis H-test + the Mann–Whitney *U* test (MW) (the nonparametric tests) depending on the results of the Levene test (indicated in Tables 2 and 3) [28, 29]. SPSS for Windows software (Release 14.0 K; SPSS Inc., Chicago, IL, USA) was used to conduct all the above-mentioned analyses. *p* < 0.05 was considered significantly different.

## 3. Results

### 3.1. Effect of PZE on AA Plus Iron-Induced Cell Death

**Figure 2:**
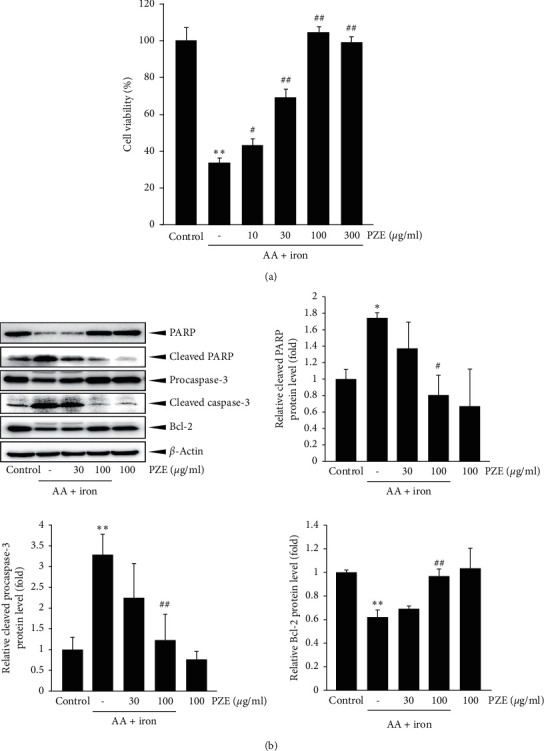
Inhibition of AA + iron-induced cell death by PZE. (a) HepG2 cells were pretreated with 10–300 *μ*g/ml PZE for 1 h and subsequently incubated with 10 *μ*M AA for 12 h, followed by exposure to 5 *μ*M iron for 4 h. Cell viability was measured by MTT assay. (b) Western blotting of apoptosis proteins was performed using HepG2 cell lysates incubated with 30 and 100 *μ*g/ml PZE for 1 h, continuously treated with 10 *μ*M AA for 12 h, and then exposed to 5 *μ*M iron for 1 h. All data represent means ± SD of three independent experiments (^*∗∗*^*p* < 0.01, ^*∗*^*p* < 0.05 between control and AA + iron-treated cells; ^##^*p* < 0.01, ^#^*p* < 0.05 between AA + iron-treated cells with or without PZE).

The protective effect of PZE against the hepatotoxicity induced by AA + iron was examined via the MTT assay. Treatment with AA + iron produced a marked decrease in the viability of HepG2 cells (*p* < 0.01). However, at the concentrations of 10–300 *μ*g/ml, PZE significantly increased the cell viability (*p* < 0.05 : 10 *μ*g/ml; *p* < 0.01 : 30, 100, and 300 *μ*g/ml) (Figure 2(a)).

### 3.2. Effect of PZE on Apoptosis-Related Protein Expressions

The protein levels of cleaved PARP, cleaved caspase-3, and Bcl-2 were analyzed by Western blot. Treatment with AA + iron increased the protein levels of cleaved PARP and cleaved caspase-3 (*p* < 0.05: cleaved PARP; *p* < 0.01: cleaved caspase-3) but reduced the protein level of Bcl-2 (*p* < 0.01). However, these changes were reversed by 100 *μ*g/ml PZE treatment (*p* < 0.05: cleaved PARP; *p* < 0.01: cleaved caspase-3 and Bcl-2) (Figure 2(b)).

### 3.3. Effect of PZE on AA + Iron-Induced Oxidative Stress and Mitochondrial Dysfunction

**Figure 3:**
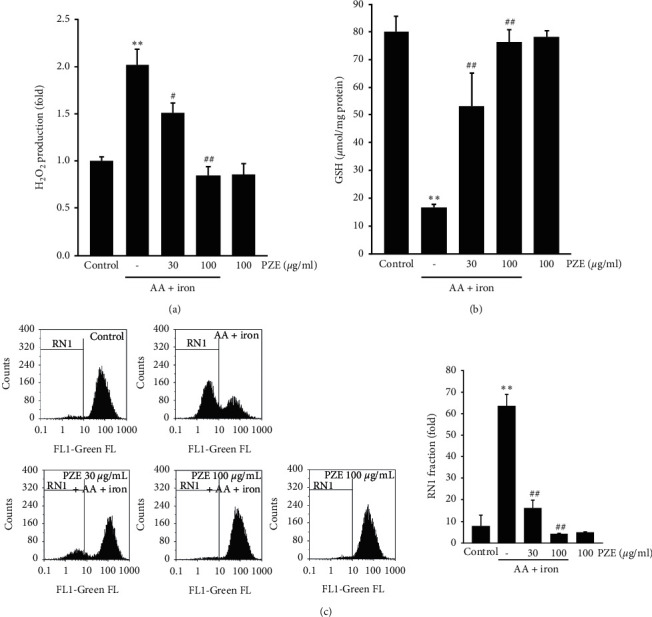
Effects of PZE on AA + iron-stimulated oxidative stress and mitochondrial dysfunction. (a) H_2_O_2_ production. (b) GSH contents. HepG2 cells were treated with 30 and 100 *μ*g/ml PZE for 12 h and then continuously exposed to 10 *μ*M AA for 12 h followed by exposure to 5 *μ*M iron for 4 h After incubation, the GSH contents were measured in cell homogenates. (c) Mitochondrial membrane permeability was measured in HepG2 cells, pretreated with 30 and 100 *μ*g/ml PZE for 1 h, and exposed subsequently to AA and iron. The cell population exhibiting low Rh123 staining intensity is represented as a fold. All data are presented as means ± SD of three independent experiments (^*∗∗*^*p* < 0.01 between control and AA + iron-treated cells; ^##^*p* < 0.01, ^#^*p* < 0.05 between AA + iron-treated cells with or without PZE).

Treatment with AA + iron increased the level of H_2_O_2_ in HepG2 cells (*p* < 0.01); however, PZE (30 and 100 *μ*g/ml) significantly inhibited the H_2_O_2_ production (30 *μ*g/ml, *p* < 0.05; 100 *μ*g/ml, *p* < 0.01) (Figure 3(a)). In addition, AA plus iron treatment reduced the intracellular level of GSH in HepG2 cells (*p* < 0.01), but pretreatment with PZE reversed this reduction (30 and 100 *μ*g/ml, *p* < 0.01) (Figure 3(b)). Moreover, AA plus iron treatment increased the population of Rh123-negative cells (RN1 fraction) (*p* < 0.01), which was inhibited by both 30 (*p* < 0.01) and 100 *μ*g/ml PZE (*p* < 0.01) (Figure 3(c)). PZE alone did not affect the H_2_O_2_ production, GSH levels, and mitochondrial function.

### 3.4. Effect of PZE on Nuclear Nrf2 Accumulation and Cytosol GCLC and NQO1 Protein Expressions

**Figure 4:**
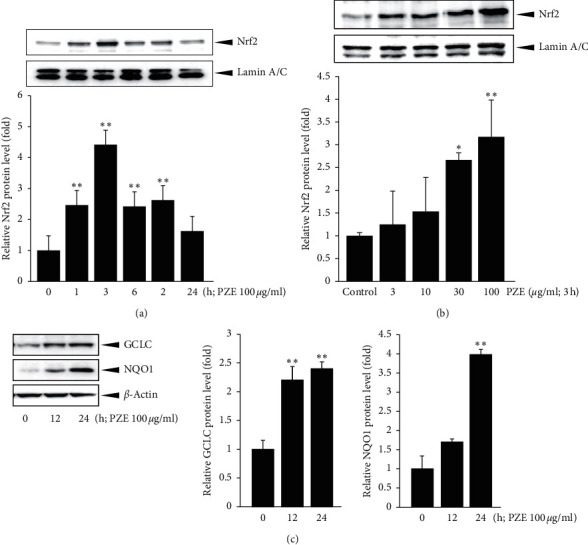
Effect of PZE on the Nrf2 nuclear translocation and the GCLC and NQO1 expressions. Western blot analysis for (a) a time (1–24 h) and (b) dose (3–100 *μ*g/ml) response on nuclear translocation of Nrf2. HepG2 cells were incubated with 100 *μ*g/ml PZE for 1–24 h or 3–100 *μ*g/ml PZE for 3 h. Equal protein loadings among the samples were verified by lamin A/C immunoblotting. The relative levels of protein bands were measured by scanning densitometry. (c) Immunoblot analysis for GCLC and NQO1. HepG2 cells were treated with 100 *μ*g/ml PZE for the indicated time period. Equal protein loadings among the samples were verified by *β*-actin immunoblotting. The relative levels of protein bands were measured by scanning densitometry. Data represent means ± SD of three separated experiments (^*∗∗*^*p* < 0.01, ^*∗*^*p* < 0.05, significant as compared with control cells).

To examine the effect of PZE on Nrf2 activation, the nuclear Nrf2 content was measured by Western blot analysis. Nuclear accumulation of Nrf2 increased significantly 1 h after treatment with 100 *μ*g/ml PZE (*p* < 0.01) and peaked at 3 h after the treatment (*p* < 0.01) (Figure 4(a)). Furthermore, treatment with 3–100 *μ*g/ml PZE for 3 h escalated the nuclear translocation of Nrf2 (*p* < 0.05 : 30 *μ*g/ml; *p* < 0.01 : 100 *μ*g/ml) (Figure 4(b)). Moreover, treatment with 100 *μ*g/ml PZE elevated the protein expressions of both GCLC (for 12 and 24 h, *p* < 0.01) and NQO1 (for 24 h, *p* < 0.01) in HepG2 cells, indicating enhanced expressions of the Nrf2-target genes (Figure 4(c)).

### 3.5. Effect of PZE on Nrf2 and ERK Phosphorylation as well as ARE-Driven Luciferase Activity

**Figure 5:**
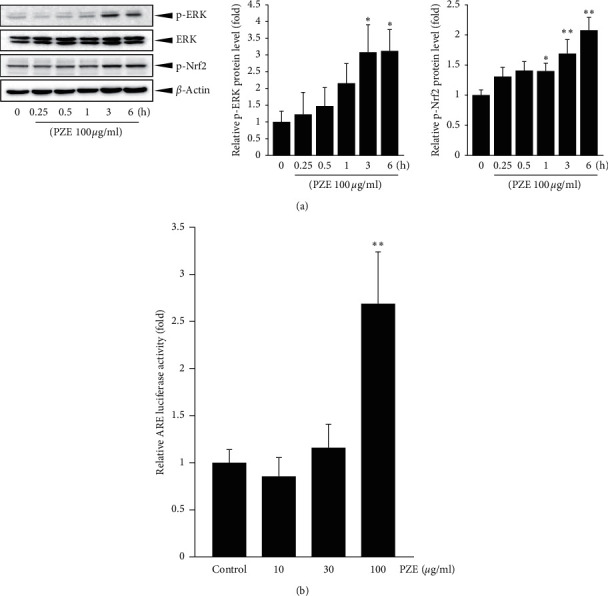
Effect of PZE on phosphorylation of ERK and Nrf2 and ARE luciferase activity. (a) Western blot analysis for phosphorylation of ERK and Nrf2. HepG2 cells were treated with 100 *μ*g/ml PZE for the indicated time period. Equal protein loadings among the samples were verified by ERK and *β*-Actin immunoblotting. The relative levels of protein bands were measured by scanning densitometry. (b) Antioxidant response element- (ARE-) driven luciferase assay. HepG2 cells stably transfected with pGL4.37 were pretreated with 10–100 *μ*g/ml PZE for 24 h, and luciferase activity in the cell lysates was subsequently measured. Data represent means ± SD of three separated experiment (^*∗∗*^*p* < 0.01, ^*∗*^*p* < 0.05, significant as compared with control cells).

Treatment with 100 *μ*g/ml PZE for 3 h or 6 h increased the expression of phospho-ERK in the cytosol of HepG2 cells (*p* < 0.05) (Figure 5(a)). In addition, PZE (100 *μ*g/ml) also increased the phospho-Nrf2 level in the cytosol, which peaked at 6 h (*p* < 0.05 for 1 h; *p* < 0.01 for 3 or 6 h) (Figure 5(a)). The functional role of Nrf2 is reflected by the ARE-driven reporter gene expression; as seen in Figure 5(b), treatment with 100 *μ*g/ml PZE for 24 h significantly increased the luciferase activity of the ARE reporter construct (*p* < 0.01).

### 3.6. Effect of PZE on Plasma ALT and AST Levels

**Figure 6:**
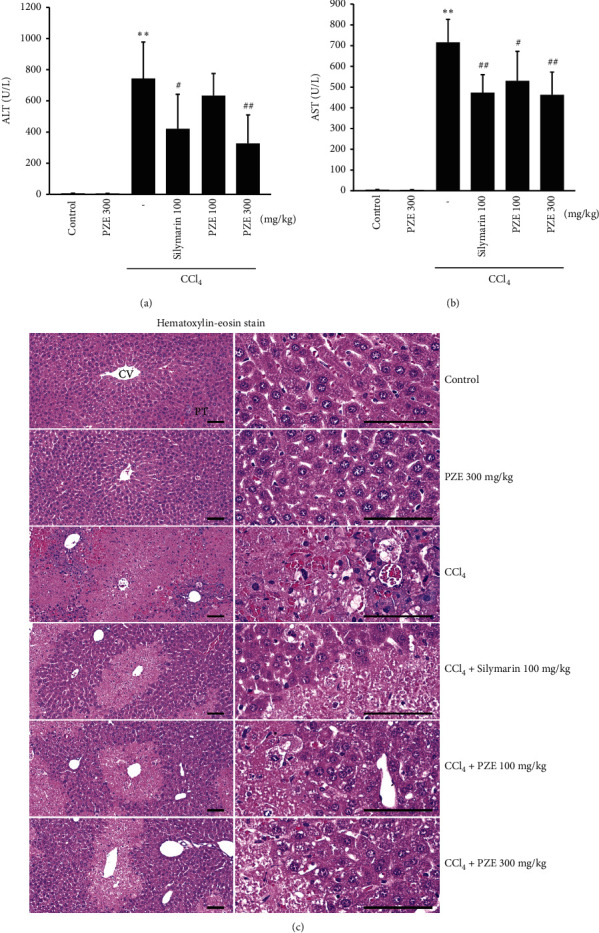
Effects of PZE on CCl_4_-induced liver injury. (a), (b) The activities of ALT and AST in blood. (c) The representative general histopathological profiles of the hepatic tissues, taken from intact or acute CCl4-treated mouse with/without PZE administration (CV: central vein; PT: portal triad regions; scale bars = 120 *μ*m). Data represent means ± SD (^*∗∗*^*p* < 0.01 compared with the vehicle-treated group; ^##^*p* < 0.01, ^#^*p* < 0.05 compared with the CCl4-treated group).

CCl_4_ administration increased the activities of plasma ALT (*p* < 0.01) and AST (*p* < 0.01). However, as seen in Figures 6(a) and 6(b), oral treatment with both 100 and 300 mg/kg PZE inhibited these increases, ALT (*p* < 0.01 for 300 mg/kg PZE) and AST (*p* < 0.05 for 100 mg/kg PZE; *p* < 0.01 for 300 mg/kg PZE). The effects did not show a dose-dependent manner, and PZE (300 mg/kg) alone did not have any impact. The positive control drug SIL at the dose of 100 mg/kg also inhibited plasma ALT and AST activities (*p* < 0.05 for ALT; *p* < 0.01 for AST), and the efficacy is comparable to that of PZE 100–300 mg/kg.

### 3.7. Effect of PZE on CCl4-Induced Histopathological Changes

As shown in Table 2 and Figure 6(c), the CCl_4_ administration increased the HAI of the liver (*p* < 0.01), the percentage of degenerative regions (*p* < 0.01), the number of degenerative hepatocytes (*p* < 0.01), and the number of infiltrated inflammatory cells (*p* < 0.01) in the liver tissue (*p* < 0.01). However, 100 mg/kg PZE (*p* < 0.01), 300 mg/kg PZE (*p* < 0.01), and 100 mg/kg SIL (*p* < 0.01) all significantly abated these increases. In addition, in the group of 300 mg/kg PZE alone, no significant changes were observed.

**Figure 7:**
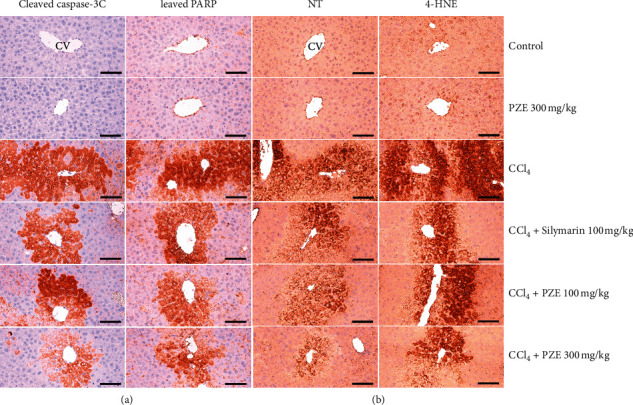
Representative (a) cleaved caspase-3, cleaved PARP, (b) NT, and 4-HNE immunoreactivities of the hepatic tissues, taken from intact or acute CCl4-treated mouse with/without PZE administration. Scale bars indicate 120 *μ*m (CV: central vein).

In the immunohistochemical analysis, as seen in Table 3 and Figure 7, there were significant immunoreactivity elevations of cleaved caspase-3 (*p* < 0.01), cleaved PARP (*p* < 0.01), NT (*p* < 0.01), and 4-HNE (*p* < 0.01) in the liver samples of the CCl_4_-treated group compared to the vehicle control group. However, treatment with 100 mg/kg PZE (*p* < 0.05 or *p* < 0.01), 300 mg/kg PZE (*p* < 0.01), or 100 mg/kg SIL (*p* < 0.01), respectively, ameliorated these histopathological changes. Once again, 300 mg/kg PZE alone did not produce any significant effects.

## 4. Discussion

PZE has been widely used in traditional Oriental medicine for treating the disorders related to the digestive system [15, 16]. In laboratory studies, PZE also displays antiparasitic, antibacterial, anti-inflammatory, and antioxidative properties [19–22]. However, no preclinical studies have ever been done to evaluate a possible therapeutic effect of PZE on hepatotoxicity. Accordingly, the present study addressed this issue in both *in vitro* and *in vivo* ways.

In *in vitro* experiments, the present study found that PZE treatment at the doses of 10–300 *μ*g/ml promoted the cell viability of AA plus iron-stimulated HepG2 cells and improved the profile of oxidative stress and the expressions of factors related to apoptosis, which were associated with enhanced activities of Nrf2 and ERK signaling pathways. In *in vivo* experiments, oral treatment with PZE at doses of 100 and 300 mg/kg/day improved the abnormal leakage of hepatic enzymes and the histopathological changes in the liver induced by CCl_4_. Taken together, these results suggest that PZE has hepatoprotective effects by counteracting oxidative stress, which may involve the ERK/Nrf2/ARE pathway.

Previously, we reported that application of AA + iron resulted in the death of hepatocytes, which is associated with oxidative stress and apoptosis [30–33]. Consistently, in the present study, there was decreased cell viability in AA plus iron-stimulated HepG2 cells; however, PZE treatment effectively rescued the cell death, indicating a possible hepatoprotective effect of PZE. Meanwhile, in the present study, AA + iron treatment overproduced H_2_O_2_, depleted the reduced GSH pool, and disturbed mitochondrial function in the hepatocytes. H_2_O_2_ is a typical marker molecule of oxidative stress; the pool of reduced GSH constitutes an important defensive line against oxidative stress; and the mitochondria are the major resource of free radical molecules in cytosol. However, in the present study, these oxidative stress-related phenomena were greatly improved by treatment with PZE. These results are in agreement with the studies reporting that PZE scavenged hydroxyl free radicals and inhibited lipid peroxidation *in vitro* [21, 22], hence suggesting that the hepatoprotection by PZE is mediated via its antioxidant action.

The increased apoptotic process is critical in the death of hepatocytes induced by AA plus iron, and both elevated oxidative stress and dysregulated equilibrium of intracellular cations contribute to this process. For example, AA has been shown to elevate the intracellular Ca^2+^ concentration and stimulate mitochondrial Ca^2+^ uptake, leading to a compromised mitochondrial function [5, 31]. The dysfunction of mitochondria induces the release of cytochrome *c*; subsequently stimulates the downstream caspases, such as caspase-3 and caspase-9; and further activates other apoptotic cellular proteins, such as PARP, finally resulting in apoptosis. In the present study, the Western blot analysis revealed that AA plus iron simulation enhanced the protein expressions of cleaved PARP and caspase-3 but reduced the protein level of Bcl-2 (an antiapoptotic protein), indicating the promoted process of apoptosis in the HepG2 cells. However, the same Western blot analysis also found that treatment with PZE effectively reversed the expression of these proteins. Although it is not clear whether these antiapoptotic effects of PZE come from possible modulation of the cellular Ca^2+^ equilibrium, from the antioxidant action, or from their combination, these results suggest that the protective effect of PZE on HepG2 cells against AA + iron is accomplished by curbing the apoptotic process.

After being separated from Keap1, Nrf2 is translocated to the nucleus and heterodimerizes with the small protein called Maf to activate the Nrf2-driven genes transcription via ARE in the promoter region [34, 35]. The Nrf2/ARE/target genes constitute an important pathway to facilitate the cellular antioxidant action because the Nrf2 target genes serve as cellular detoxicants, antioxidants, and ROS scavengers [36]. In the present study, PZE treatment significantly increased the nuclear accumulation of Nrf2 in HepG2 cells; consequently, we can consider that PZE activates the Nrf2/ARE pathway to exert its antioxidant action. This idea is directly supported in the present study by the result that PZE treatment markedly raised the ARE luciferase activity in the HepG2 cells, which reflects the increased activation of the Nrf2/ARE/target genes pathway, and is also reinforced by the result that PZE elevated the expressions of antioxidant proteins GCLC and NQO1 in the HepG2 cells, which are both the Nrf2 target genes' production [37–39]. It has been well documented that the phosphorylation of Nrf2 at serine 40 by ERK1/2 facilitates the dissociation of Nrf2 with Keap1 and promotes the nuclear translocation of Nrf2 [40, 41]. In the present study, treatment with 100 *μ*g/ml PZE for 3–6 h significantly increased the phosphorylation rates of ERK in HepG2 cells, and the same dose of PZE for 1–6 h also escalated the phosphorylation rates of Nrf2 by the similar fashion with the case of ERK. These results suggest that phosphorylation of Nrf2 by the MAPK pathway contributes to the activation of Nrf2 by PZE. Altogether, these results collectively suggest that the hepatoprotective effects of PZE against AA + iron-induced oxidative stress are mediated via the ERK/Nrf2/ARE pathway.

CCl_4_ generates substantial damage to liver. ALT and AST are both typical biochemical markers of hepatocytic injury, leaked from injured liver cells to enter into the blood [42]. In the present study, the CCl_4_ administration significantly increased plasma ALT and AST levels in mice. However, the levels of the ALT and AST were significantly reduced by treatment with 300 mg/kg PZE, and 100 mg/kg PZE also inhibited the increase of plasma AST levels. SIL, a flavonoid included in the herb milk thistle, *Silybum marianum*, represents a typical herb-origin antioxidant that has hepatoprotective effects [43]. In the present study, SIL (100 mg/kg/day) was employed to be a positive pharmacological control, and the oral administration of SIL also significantly inhibited CCl_4_-induced increases of plasma ALT and AST activity. These results biochemically suggest that PZE protects the liver against CCl_4_-induced toxicity *in vivo*.

Consistent with the previous reports [23, 24], in the present study, the histopathological observation revealed that there was vacuolation (deposition of lipid droplets) and ballooning of hepatocytes, as well as inflammatory cell infiltration in CCl_4_-treated mice. The damaged hepatocytes were mainly distributed around central veins, and cells with fatty changes were found in the margins. However, in the present study, treatment with both 100 and 300 mg/kg PZE doses, respectively, attenuated the heightened HAI index (denoting the severity of hepatic tissue damage, see Tables 1 and 2), the elevated percentages of degenerated regions, the increased numbers of degenerated hepatocytes, and the enhanced numbers of infiltrated inflammatory cells in the hepatic tissues induced by CCl_4_ administration. These results are in agreement with the biochemical results and histologically suggest that PZE produces protective effects against CCl_4_-induced liver injury.

As seen in the *in vitro* experiments, the increased hepatic apoptosis appears to be the major cause of AA + iron-induced hepatocytic death [30], and this holds true for the *in vivo* experiment [44]. The immunohistochemical analysis in the present study revealed that there were increased numbers of cleaved caspase-3 immunoreactive cells and cleaved PARP immunoreactive cells in the CCl_4_-treated mice liver, which were mainly located in centrolobular regions. However, these increased immunoreactivities were greatly reduced by treatment with 100 and 300 mg/kg PZE as well as 100 mg/kg SIL. These results lend strong support to the results of the *in vitro* experiment that evaluated the same apoptotic molecules and histologically confirm the inhibitory effect of PZE on toxicants-induced hepatocytic apoptosis.

NT is a tyrosine nitration product that is generated by reactive nitrogen species including peroxynitrite anions and nitrogen dioxide [45, 46], and 4-HNE is an *α*,*β*-unsaturated hydroxyalkenal yielded by cell lipid peroxidation [47, 48]. In the present study, obvious increases in the number of NT and 4-HNE immunoreactive cells were immunohistochemically observed in the hepatic tissues of CCl_4_-treated mice; however, they were, respectively, reversed by 100 mg/kg PZE, 300 mg/kg PZE, or 100 mg/kg SIL treatment. These findings not only immunohistochemically present the oxidative stress mechanism of CCl_4_-induced hepatotoxicity, but also consolidate the *in vitro* evidence that PZE protects the hepatocytes against oxidative stress.

In summary, in the present study, PZE *in vitro* and *in vivo* exerted protective effects against AA plus iron-induced hepatocytotoxicity and carbon tetrachloride-induced liver injury. Besides, PZE *in vitro* improved the status of oxidative stress and normalized the protein expressions of apoptosis-related factors, which were also observed *in vivo.* Meanwhile, PZE boosted nuclear translocation of Nrf2, increased ARE luciferase activity, and elevated the phosphorylation rates of Nrf2 and ERK, reflecting the promoted activity of the ERK/Nrf2/ARE pathway. These findings collectively suggest that PZE has protective effects against xenotoxicants-induced hepatotoxicity by antagonizing increased oxidative stress via the ERK/Nrf2/ARE pathway, and direct us toward future investigation for identifying the bioactive compounds that account for the hepatoprotection by PZE.

## Figures and Tables

**Table 1:** Modified HAI grading: inflammatory scores used in the present study.

a. Confluent necrosis	
Absent	0
Focal confluent necrosis	1
Zone 3 necrosis in some areas	2
Zone 3 necrosis in most areas	3
Zone 3 necrosis + occasional portal-central bridging	4
Zone 3 necrosis + multiple portal-central bridging	5
Panacinar or multiacinar necrosis	6

b. Focal (spotty) lytic necrosis, apoptosis, and focal inflammation	
Absent	0
One focus or less per 10 × objective	1
Two to four foci per 10 × objective	2
Five to ten foci per 10 × objective	3
More than ten foci per 10 × objective	4

HAI: histological activity index, HAI grading scores = *A* + B, possible maximum total scores = 10, modified from the method described by Ishak et al. [25].

**Table 2:** General histomorphometrical analysis of acute CCl_4_-treated mouse hepatic tissues.

Groups	General histomorphometry			
	Histological activity index (scores; max = 10)	Percentages of degenerative regions (%/mm^2^)	Numbers of degenerative hepatocytes (cells/1000 hepatocytes)	Numbers of inflammatory cells infiltrated (cells/mm^2^)
Control	0.40 ± 0.55	1.54 ± 0.78	14.80 ± 7.95	24.20 ± 6.98
PZE 300	0.40 ± 0.55	1.53 ± 0.87	15.00 ± 7.78	23.80 ± 12.64
CCl_4_	8.20 ± 1.10^a^	83.37 ± 6.10^c^	808.80 ± 78.10^c^	223.00 ± 76.42^c^
CCl_4_ + silymarin	4.40 ± 0.55^ab^	47.02 ± 4.58^ce^	453.20 ± 50.01^ce^	71.60 ± 10.29^ce^
CCl_4_ + PZE 100	4.60 ± 0.89^ab^	48.11 ± 7.95^ce^	473.60 ± 86.38^ce^	71.00 ± 14.09^ce^
CCl_4_ + PZE 300	3.40 ± 0.55^ab^	38.55 ± 6.63^ce^	363.80 ± 59.82^ce^	51.00 ± 15.22^de^

^a^ *p* < 0.01 as compared with control by LSD test, ^b^*p* < 0.01 as compared with CCl_4_ by LSD test, ^c^*p* < 0.01 and ^d^*p* < 0.05 as compared with control by MW test, ^e^*p* < 0.01 as compared with CCl_4_ by MW test.

**Table 3:** Immunohistochemical-histomorphometrical analysis of acute CCl_4_-treated mouse hepatic tissues.

Groups	Positive cells by immunohistochemistry (cells/1000 hepatocytes)			
	Cleaved caspase-3	Cleaved poly (ADP-ribose) polymerase	Nitrotyrosine	4-Hydroxynonenal
Control	12.20 ± 9.18	19.80 ± 10.94	28.40 ± 18.22	62.40 ± 30.66
PZE 300	12.00 ± 7.65	19.40 ± 9.66	27.20 ± 20.75	61.40 ± 37.58
CCl_4_	664.60 ± 77.89^c^	713.40 ± 57.50^c^	664.20 ± 50.00^c^	795.60 ± 60.11^a^
CCl_4_ + silymarin	420.00 ± 33.13^cd^	463.80 ± 57.12^cd^	388.40 ± 69.68^cd^	404.40 ± 51.03^ab^
CCl_4_ + PZE 100	433.20 ± 89.18^cd^	461.20 ± 77.33^cd^	412.80 ± 133.19^ce^	427.20 ± 83.58^ab^
CCl_4_ + PZE 300	261.80 ± 50.98^cd^	284.00 ± 108.91^cd^	217.20 ± 37.10^cd^	278.60 ± 45.39^ab^

^a^ *p* < 0.01 as compared with control by LSD test, ^b^*p* < 0.01 as compared with CCl_4_ by LSD test, ^c^*p* < 0.01 as compared with control by MW test, ^d^*p* < 0.01 and ^e^*p* < 0.05 as compared with CCl_4_ by MW test.

## Data Availability

The data justifying the conclusions of this study are all statistically analyzed and presented in the Results section and are also available from the corresponding authors.
